# Knowledge-driven binning approach for rare variant association analysis: application to neuroimaging biomarkers in Alzheimer’s disease

**DOI:** 10.1186/s12911-017-0454-0

**Published:** 2017-05-18

**Authors:** Dokyoon Kim, Anna O. Basile, Lisa Bang, Emrin Horgusluoglu, Seunggeun Lee, Marylyn D. Ritchie, Andrew J. Saykin, Kwangsik Nho

**Affiliations:** 1 Biomedical & Translational Informatics Institute, Geisinger Health System, Danville, PA USA; 2 The Huck Institutes of the Life Sciences, Pennsylvania State University, University Park, PA USA; 3 Department of Biostatistics and Center for Statistical Genetics, University of Michigan, Ann Arbor, MI USA; 4 Center for Neuroimaging, Department of Radiology and Imaging Sciences, Indiana University School of Medicine, Indianapolis, IN USA

**Keywords:** Rare variant analysis, Imaging genomics, Alzheimer’s disease

## Abstract

**Background:**

Rapid advancement of next generation sequencing technologies such as whole genome sequencing (WGS) has facilitated the search for genetic factors that influence disease risk in the field of human genetics. To identify rare variants associated with human diseases or traits, an efficient genome-wide binning approach is needed. In this study we developed a novel biological knowledge-based binning approach for rare-variant association analysis and then applied the approach to structural neuroimaging endophenotypes related to late-onset Alzheimer’s disease (LOAD).

**Methods:**

For rare-variant analysis, we used the knowledge-driven binning approach implemented in Bin-KAT, an automated tool, that provides 1) binning/collapsing methods for multi-level variant aggregation with a flexible, biologically informed binning strategy and 2) an option of performing unified collapsing and statistical rare variant analyses in one tool. A total of 750 non-Hispanic Caucasian participants from the Alzheimer’s Disease Neuroimaging Initiative (ADNI) cohort who had both WGS data and magnetic resonance imaging (MRI) scans were used in this study. Mean bilateral cortical thickness of the entorhinal cortex extracted from MRI scans was used as an AD-related neuroimaging endophenotype. SKAT was used for a genome-wide gene- and region-based association analysis of rare variants (MAF (minor allele frequency) < 0.05) and potential confounding factors (age, gender, years of education, intracranial volume (ICV) and MRI field strength) for entorhinal cortex thickness were used as covariates. Significant associations were determined using FDR adjustment for multiple comparisons.

**Results:**

Our knowledge-driven binning approach identified 16 functional exonic rare variants in *FANCC* significantly associated with entorhinal cortex thickness (FDR-corrected *p*-value < 0.05). In addition, the approach identified 7 evolutionary conserved regions, which were mapped to *FAF1*, *RFX7*, *LYPLAL1* and *GOLGA3*, significantly associated with entorhinal cortex thickness (FDR-corrected *p*-value < 0.05). In further analysis, the functional exonic rare variants in *FANCC* were also significantly associated with hippocampal volume and cerebrospinal fluid (CSF) Aβ_1–42_ (*p*-value < 0.05).

**Conclusions:**

Our novel binning approach identified rare variants in *FANCC* as well as 7 evolutionary conserved regions significantly associated with a LOAD-related neuroimaging endophenotype. *FANCC* (fanconi anemia complementation group C) has been shown to modulate TLR and p38 MAPK-dependent expression of IL-1β in macrophages. Our results warrant further investigation in a larger independent cohort and demonstrate that the biological knowledge-driven binning approach is a powerful strategy to identify rare variants associated with AD and other complex disease.

## Background

Rapid advances in next-generation sequencing technologies and bioinformatics tools over the past decade have made an important contribution to searching for disease susceptibility factors and understanding the impact of the genetic variation on human diseases [1, 2]. In particular, since the completion of the human genome project, whole genome sequencing (WGS) has been increasingly used as a tool to understand the complexity and diversity of genomes in disease by performing detailed evaluation of all genetic variation [3, 4].

Late-onset Alzheimer’s disease (LOAD) is the most prevalent form of age-related neurodegenerative disease and dementia [5]. Abnormal proteins forming histologically visible structures, amyloid plaques and neurofibrillary tangles, damage and destroy neurons and their connections [6]. With the increasing population of aging adults, it is predicted that the number of AD patients will triple in the United States by 2050 [7]. Models suggest that delaying the onset of AD by 5 years through early intervention could reduce the number of AD cases by nearly 50% [8, 9]. To develop effective therapeutic intervention to slow or prevent disease progression and to effectively target potential disease-modifying approaches, early biomarkers are needed to detect AD at pre-symptomatic stages with high accuracy and monitor the pathological progression. With an estimated heritability of about 80%, genetic factors play an important role in developing AD [10, 11]. Very recently, genetic association studies have used next-generation sequencing technologies to identify functional risk rare variants with moderate to large effects on LOAD risk within *TREM2, ABCA7*, *UNC5C*, *AKAP9* and *PLD3* genes [12–14].

For a rare-variant association analysis, gene- or region-based multiple-variant tests have been widely used due to improved power over single variant tests. There exist several different approaches in multiple-variant tests. Burden methods test the cumulative effect of variants within a knowledge-driven region such as genes and are easily applied to case–control studies as they assess the frequency of variant counts between these binary phenotypes. Burden tests, which collapse variants to a single genetic score, are powerful when the variants have the same effect direction with similar magnitudes [15]. When this assumption is violated, however, it can result in a significant loss of power. Variance component tests, such as sequence kernel association test (SKAT), were developed to overcome this limitation [16]. SKAT is a score-based variance component test that uses a multiple regression kernel-based approach to assess variant distribution and test for association. These are more powerful than Burden tests in the presence of opposite association directions or large numbers of non-causal variants [16].

A rare-variant study requires careful consideration, including choice of variant collapsing or binning approach for region-based association analysis. In this study, we propose a novel biological knowledge-driven binning approach (Bin-KAT) to identify trait- and disease-associated rare variants. Bin-KAT is a comprehensive, streamlined approach that unifies a genome-wide variant binning function in BioBin [17–21] and a dispersion-based association analysis tool such as SKAT [16, 22].

## Methods

### Study subjects and whole genome sequencing (WGS) analysis

This study utilized data from the Alzheimer’s Disease Neuroimaging Initiative (ADNI) cohort. The ADNI cohort consisted of cognitively normal older adults (CN), mild cognitive impairment (MCI) and early AD. We downloaded demographic information, raw MRI scan data, whole genome sequencing data and diagnostic information from the ADNI data repository (http://www.loni.usc.edu/ADNI/) [23]. All participants provided written informed consent and study protocols were approved by each participating sites’ Institutional Review Board. WGS was performed by Illumina on blood-derived genomic DNA samples obtained from 818 ADNI participants using paired-end 100-bp reads on the Illumina HiSeq2000 (www.illumina.com). As described previously in detail [24, 25], Broad GATK and BWA-mem were used to align raw sequence data to the reference human genome (human genome build 37) and call the variants.

### Neuroimaging analysis

All available structural MRI scans at baseline acquired following the ADNI MRI protocol were downloaded from the ADNI data repository [26]. A widely employed automated MRI analysis technique, FreeSurfer (http://surfer.nmr.mgh.harvard.edu/), for automated segmentation and parcellation, was used to process MRI scans and extract mean volumes and cortical thicknesses (Euclidean distance between the grey/white boundary and the grey/cerebrospinal fluid boundary) for all target regions. In this analysis, we used the bilateral mean value of the entorhinal cortex thickness as an AD-related endophenotype as the entorhinal cortex is a region known to be affected early in AD.

### Knowledge-driven binning approach

**Fig. 1:**
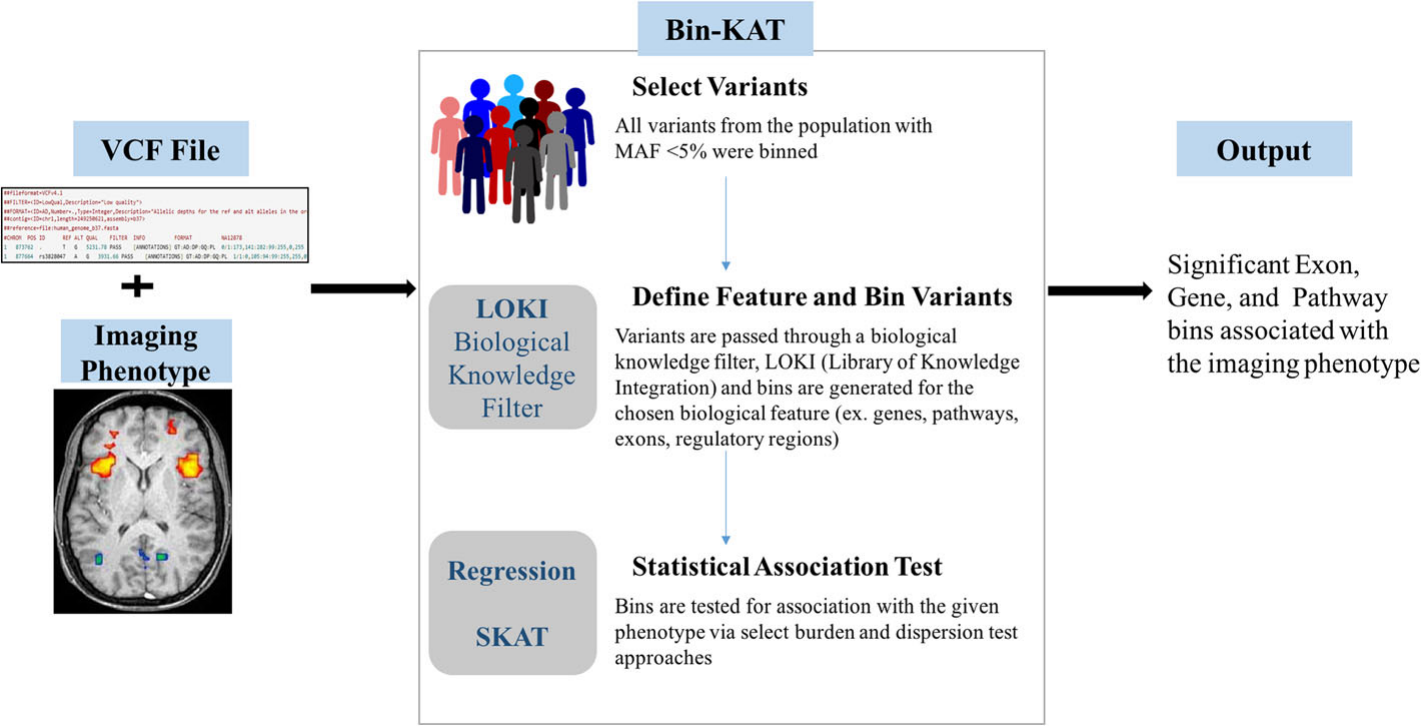
Illustration of rare variant association analysis using Bin-KAT for neuroimaging genomics. First, rare variants were binned/collapsed based on biological knowledge, such as exon, gene, pathway, protein family, evolutionary conversed regions (ECR) or regulatory region, using BioBin. Then, statistical tests including a burden test and a dispersion test (SKAT), were incorporated into BioBin, called Bin-KAT [19]. Bin-KAT provides an option of performing unified rare variant association analysis methods in one tool to identify biologically-informed bins significantly associated with imaging endophenotypes of interest. VCF, variant call format

As a variant binning tool, BioBin aggregates variants into multiple user-selected features in a biologically informed manner using an internal biological data repository known as LOKI or the Library of Knowledge Integration. LOKI integrates multiple public databases including NCBI Entrez Gene, UCSC Genome Browser, Protein families (Pfam), Kyoto Encyclopedia of Genes and Genomes (KEGG), Reactome, Genome Ontology (GO) and others, into one centralized data bank. Using these rich data sources, variants can be binned into various biological features such as genes, pathways, protein families, evolutionary conserved regions (ECRs), regulatory regions and others. The main utility of BioBin is a direct access to a comprehensive knowledge-guided binning approach for multiple biological features. Simultaneous to variant binning, a user can perform a phenotypic association analysis using selected burden tests (regression or the Wilcoxon rank sum) or dispersion tests (SKAT) directly within the framework of BioBin. Our knowledge-driven binning approach (Bin-KAT) was applied to determine the association of rare variants with LOAD-related neuroimaging endophenotype, entorhinal cortex thickness (Fig. 1), while adjusting for age, gender, years of education, intracranial volume (ICV) and MRI field strength. Functional exonic rare variants (minor allele frequency (MAF) < 0.05) extracted from the WGS data using ANNOVAR [27] were binned by five different biological features, genes, KEGG pathway, protein families, regulatory regions and ECRs (Fig. 1). A minimum bin size of 5 variants was used. Binned variants were weighted inversely proportional to their MAF using Madsen and Browning weighting [28].

## Results

### Genome-wide gene-based association analysis of functional exonic rare variants with LOAD-related neuroimaging endophenotype

**Fig. 2:**
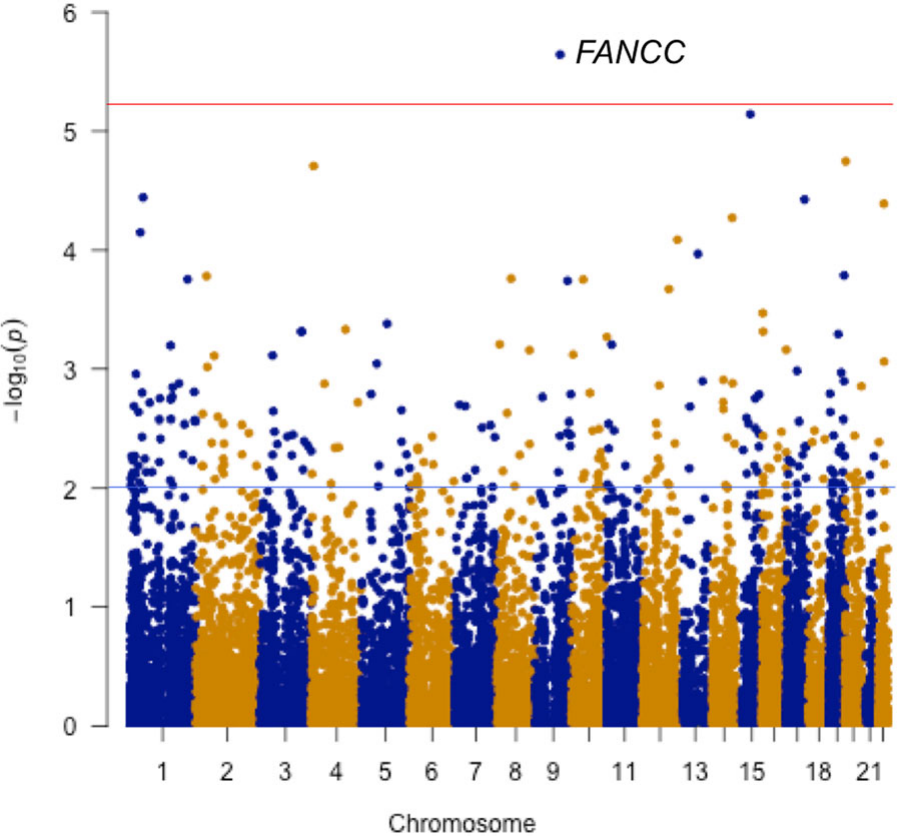
Manhattan plot of genome-wide gene-based rare variant association analysis for a LOAD-related neuroimaging endophenotype, entorhinal cortex thickness. –log_10_ *p*-value was plotted against the chromosomal location of each gene. *FANCC* exceeded the genome-wide significant threshold (FDR-corrected *p*-value = 0.05) (*red line*)

In order to remove spurious association in disease studies due to population stratification, a total of 750 non-Hispanic Caucasian ADNI participants who had both WGS data and MRI scans were used in this study [29]. The population demographics are shown in Table 1. From the WGS-identified variants, ANNOVAR identified 205,136 functional exonic variants. Among 205,136 variants, 188,508 rare variants (MAF < 0.05) were selected for the analysis. A genome-wide gene-based association analysis of rare variants with entorhinal cortex thickness using a burden-based approach did not identify any genes that exceeded a genome-wide significant threshold (FDR-corrected *p*-value < 0.05) (data not shown). However, a dispersion-based approach (SKAT) identified a gene, *FANCC*, which consisted of 16 functional exonic rare variants, achieved a genome-wide significant association with entorhinal cortex thickness (*p*-value < 2 x 10^−6^; FDR-corrected *p*-value < 0.05) (Fig. 2). To further investigate the effect of rare variants in *FANCC* on phenotypic variation, we re-ran SKAT for *FANCC* after removing one variant at a time and identified that rs1800361 out of 16 variants in *FANCC* had the strongest effect on entorhinal cortex thickness (Table 2). In addition, the functional exonic rare variants in *FANCC* were also associated with hippocampal volume and cerebrospinal fluid (CSF) Aβ_1–42_ (*p*-value < 0.05).

**Table 1:** Demographic characteristics of study population

	CN	EMCI	LMCI	AD
N	255	218	232	45
Gender (M/F)	129/126	120/98	148/84	28/17
Age (mean (SD))	74.38 (5.47)	71.12 (7.46)	73.16 (7.27)	74.76 (9.25)
Education (mean (SD))	16.4 (2.7)	16.0 (2.7)	16.1 (3.0)	15.7 (2.7)
*APOE* ε4 (absence/presence)	185/70	131/87	113/119	12/33

*CN* cognitive normal older subject, *EMCI* early mild cognitive impairment, *LMCI* late MCI, *SD* standard deviation

**Table 2:** Variant effects of *FANCC* on entorhinal cortex thickness. *P*-values from SKAT were obtained by removing a rare variant on *FANCC* at a time

Variant	*p-*value^a^	Annotation
rs1800361	3.01E-04	nonsynonymous
rs145497019	1.20E-05	nonsynonymous
rs1800362	9.71E-06	nonsynonymous
rs1800368	9.71E-06	nonsynonymous
rs138629441	5.79E-06	nonsynonymous
rs143152201	3.19E-06	nonsynonymous
9:97869388	2.33E-06	nonsynonymous
*FANCC*	2.27E-06^b^	
9:97887391	2.27E-06	nonsynonymous
rs1800367	2.27E-06	nonsynonymous
9:97876956	2.20E-06	nonsynonymous
rs140687953	1.87E-06	nonsynonymous
rs140781259	1.70E-06	nonsynonymous
9:97934335	1.66E-06	nonsynonymous
rs1800366	1.65E-06	nonsynonymous
rs121917783	1.59E-06	stop-gain
rs1800365	1.48E-06	nonsynonymous

^a^ *p*-value from SKAT for *FANCC* after removing the variant

^b^ *p*-value from SKAT for *FANCC* that contains every variant

**Fig. 3:**
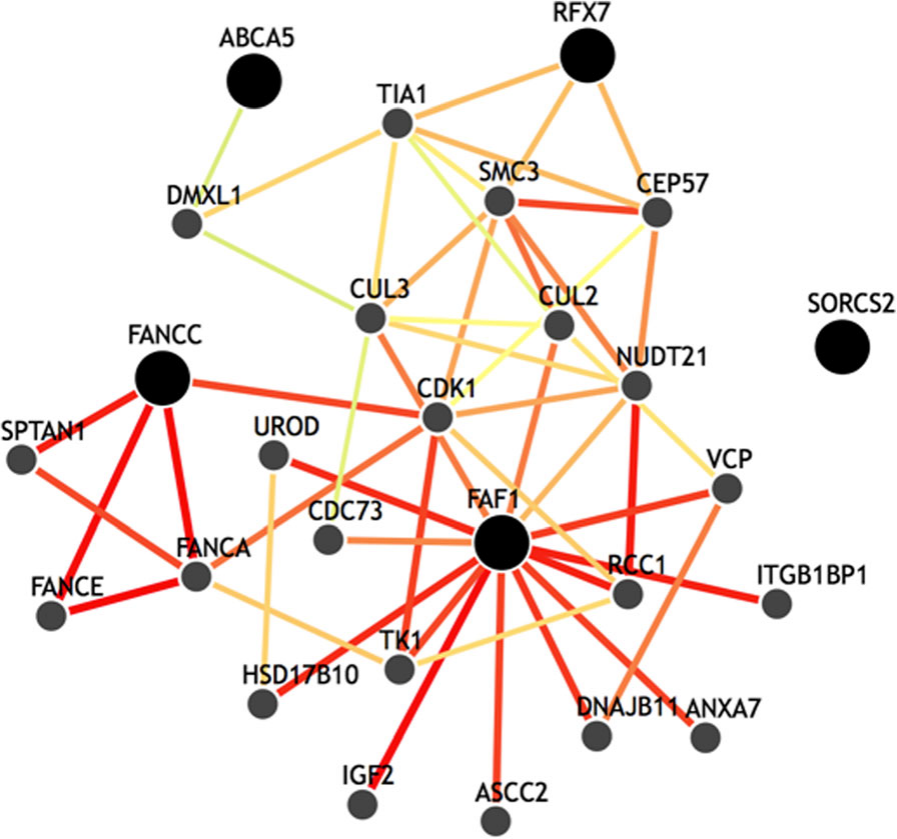
Functional networks based on top 5 genes associated with entorhinal cortex thickness. The Integrated Multi-species Prediction (IMP) performs a graphical search of a functional network to identify the genes most likely to participate in similar pathways as query genes including *FANCC*, *RFX1*, *FAF1*, *ABCA5* and *SORCS2. Nodes* represent genes and *edges* represent the predicted probability that the connected genes are involved in the same biological process. *Large nodes* represent query genes and the *color of the edge* signifies the strength of the relationship confidence. *Red edge* represents higher confidence scores between nodes

There were several genes marginally associated with entorhinal cortex thickness. Top 10 genes including *FANCC* were obtained based on SKAT *p*-values (Table 3). In particular, five genes (*RFX7*, *SORCS2*, *FAF1*, *ABCA5* and *NCF4*) were marginally significant within FDR-corrected *p*-value < 0.1 (Table 3). To identify a functional relationship between top 5 genes, we performed the Integrated Multi-species Prediction (IMP) that combines biological evidence from multiple biological databases and provides a probability score that two genes are involved in a biological and functional relationship [30]. Figure 3 shows that *FANCC*, *RFX7*, *FAF1* and *ABCA5* are likely to be involved in the same biological process.

**Table 3:** Top 10 genes associated with entorhinal cortex thickness

Gene	*p*-value	FDR-corrected *p*-value
*FANCC*	2.27E-06	**0.033**
*RFX7*	7.22E-06	0.052
*SORCS2*	1.95E-05	0.094
*FAF1*	3.60E-05	0.098
*ABCA5*	3.75E-05	0.098
*NCF4*	4.06E-05	0.098
*RIN3*	5.33E-05	0.110
*MFSD2A*	7.08E-05	0.128
*GOLGA3*	8.16E-05	0.132
*CLN5*	1.07E-04	0.156

### Knowledge-based binning approach for an association analysis of rare variants

In addition to a gene rare variant analysis approach, our biological knowledge-based binning approach based on KEGG pathway, Pfam, ECRs and regulatory regions was performed. None of biologically-informed bins was significant when the burden-based approach was used (data not shown). However, the dispersion approach (SKAT) identified 7 evolutionary conserved regions, which were mapped to *FAF1*, *RFX7*, *LYPLAL1* and *GOLGA3*, significantly associated with entorhinal cortex thickness (FDR-corrected *p*-value < 0.05) (Table 4).

**Table 4:** Evolutionary conserved regions (ECR) associated with entorhinal cortex thickness

ECR	Mapped gene	*p*-value	FDR-corrected *p*-value
ucsc_ecr:ecr_placentalMammals_chr1_band514	*FAF1*	1.72E-06	0.018
ucsc_ecr:ecr_placentalMammals_chr15_band358	*RFX7*	1.72E-06	0.018
ucsc_ecr:ecr_primates_chr15_band292	*RFX7*	5.81E-06	0.025
ucsc_ecr:ecr_vertebrate_chr1_band599	*FAF1*	7.22E-06	0.025
ucsc_ecr:ecr_vertebrate_chr1_band1917	*LYPLAL1, LOC101929666, LOC101929713*	7.22E-06	0.025
ucsc_ecr:ecr_vertebrate_chr12_band1255	*GOLGA3*	7.22E-06	0.025
ucsc_ecr:ecr_vertebrate_chr15_band428	*RFX7*	8.45E-06	0.025

## Discussion

In this study we developed a novel knowledge-driven binning approach for rare-variant association analysis and then applied the approach to whole genome sequencing data to identify rare variants associated with a neuroimaging endophenotype related to LOAD. Our results showed that (1) the novel binning approach is useful to identify trait- and disease-associated rare variants; (2) a dispersion-based test (SKAT) outperforms a regression-based burden test [19]; and (3) quantitative traits (QT) as phenotypes substantially increase detection power for association analysis.

The biological knowledge-based binning approach identified rare variants in *FANCC* (Fanconi anemia complementation group C) as well as 7 evolutionary conserved regions significantly associated with a LOAD-related neuroimaging endophenotype, entorhinal cortex thickness. The entorhinal cortex (EC) is a region that is affected early in the progression of AD and one of the first sites of tau pathology, and the entorhinal cortex thickness was shown to predict cognitive decline in AD [31, 32].

Although the relationship between Fanconi anemia (FA) genes and AD has not been identified yet, there are some genetic modulators playing a role in FA and AD pathology. FA genes include several complementation groups [33, 34]. FA proteins form the complexes with each other against genotoxic stress for the survival of the hematopoietic and germ cells [33]. In addition to playing a role in the FA complex during homologous recombination repair, *FANCC* has the other crucial function in hematopoietic cells by protecting them from apoptosis [33, 35]. *FANCC* has been shown to modulate TLR and p38 MAPK-dependent expression of IL-1β in macrophages [36]. *FANCC* −/− mice produce 2.5 times more interleukin 1β (IL-1β) than wild type and in human CD14+ cells [37]. In addition to these roles of IL-1β and MAP kinases in the FA pathway, IL-1β and p38 MAPK and JNK were significantly related to Aβ-induced EC synaptic dysfunction by involving the receptor for advanced glycation end products (RAGE) signaling in microglia in AD mice model [38]. *FANCC* binds and regulates the phosphorylation of the Stathmin-1 (STMN1) that is crucial for the spindle organization during mitosis [39]. In addition, a microarray expression study showed that STMN1 is differentially expressed in AD and associated with calcium hemostasis in the human brain [40].

The evolutionary conserved regions (ECRs) we identified to be associated with entorhinal cortex thickness were also linked to the MAPK-p38 pathway [41, 42]. The ECRs are often required for basic cellular or metabolic function; finding ECRs is a useful method for identifying functional sequences in a genome. Several ECRs were identified to be associated with entorhinal cortex thickness including *FAF1*, which was found to activate the MAPK p38 signaling pathway [43]. *FAF1* has also been found to be overexpressed in the frontal cortex of Parkinson’s disease (PD) as well as PD and AD patients [44]. *GOLGA3* (golgin A3) has been found to have upregulated expression in AD possibly by promoting cell surface expression of the beta1-adrenergic receptor [45]. *RFX7* plays an important role in the development of the neural tube during embryogenesis [46], and is highly expressed in various brain tissues [47]. Since the genes we mentioned above were related to the pathways common with AD pathology, these genes may be a potential target for future therapeutics to treat neurodegenerative disease and cognitive decline.

## Conclusions

To conclude, our results warrant further investigation in a larger independent cohort and demonstrate that the knowledge-driven binning approach using Bin-KAT is a powerful strategy to identify rare variants associated with AD and other complex disease. Bin-KAT has previously shown to be successful in a multiple phenotype and multiple biological feature analysis [19]. This software package is open source and freely available from http://ritchielab.com/software/biobin-download.
